# Acute shortening and re-lengthening versus antibiotic calcium sulfate-loaded bone transport for the management of large segmental tibial defects after trauma

**DOI:** 10.1186/s13018-022-03109-1

**Published:** 2022-04-10

**Authors:** Qiang Huang, YiBo Xu, Yao Lu, Cheng Ren, Lu Liu, Ming Li, Qian Wang, Zhong Li, HanZhong Xue, Kun Zhang, Teng Ma

**Affiliations:** Department of Orthopaedic Surgery, Hong Hui hospital, Xi’an Jiaotong University College of Medicine, Xi’an, 710054 Shaanxi China

**Keywords:** Acute shortening, Bone transport, Calcium sulfate, Tibial defect

## Abstract

**Background:**

The purpose of this paper was to compare the clinical effects of acute shortening and re-lengthening (ASR) technique with antibiotic calcium sulfate-loaded bone transport (ACSBT) technique for the management of large segmental tibial defects after trauma.

**Methods:**

In this retrospective study, 68 patients with large segmental tibial defects were included and completely followed. The bone loss was 3–10 cm. ASR group included 32 patients, while ACSBT group contained 36. There was no significant difference in demographic information between the two groups. The external fixation time (EFT) and external fixation index (EFI) were compared. Bone defect healing and limb functions were evaluated according to the Association for the Study and Application of the Method of Ilizarov (ASAMI) criteria. Complications were compared by Paley classification.

**Results:**

The mean EFT was 9.2 ± 1.8 months in ASR group and 10.1 ± 2.0 months in ACSBT group, respectively. The mean EFI was 1.5 ± 0.2 month/cm and 1.4 ± 0.3 month/cm. According to the ASAMI criteria, in ASR group bone defect healing was excellent in 22 cases, good in 7 cases and fair in 3 cases. In ACSBT group, it was excellent in 23 cases, good in 11 cases and fair in 2 cases. In ASR group, the limb function was excellent in 15 cases, good in 7 cases and fair in 10 cases, while it was excellent in 14 cases, good in 9 cases and fair in 13 cases with ACSBT group. There was no significant difference in EFI, bone defect healing and limb functions between the two groups (*p* > 0.05). The mean number of complications per patient in ACSBT group was significantly lower than that in ASR group (*p* < 0.05).

**Conclusion:**

Both techniques can be successfully used for the management of large segmental tibial defects after trauma. There was no significant difference in EFI, limb functions and bone defect healing between the two groups. Compared with ASR group, the complication incidence in ACSBT group was lower, especially the infection-related complications. Therefore, for patients with large segmental bone defects caused by infection or osteomyelitis, ACSBT technique could be the first choice.

## Background

Patients with severe open injuries or chronic osteomyelitis of the tibia are prone to large segmental tibial defects. They are often accompanied by large skin and soft tissue defects which are difficult to deal with [1–3]. Ilizarov technique is one of the most effective methods for such patients. It emphasizes the flexible use of tension–stress law and individualized selection of fixation methods [4, 5]. The commonly used Ilizarov techniques include bone transport, ASR, etc. [6].

Since its discovery, traditional bone transport technique has saved many limbs on the verge of amputation. However, this technique displays some obvious drawbacks. Local antibiotic carriers are not regularly used in traditional bone transport surgeries. Due to lack of high concentration antibiotics locally, the infection-related complications increase, such as pin-tract infection and deep infection. In addition, sclerosis and resorption occur at both ends of bone defects during transport process. This results in docking site nonunion and adds unplanned revision surgeries [7]. Therefore, several scholars developed ASR technique and used it in patients with large segmental bone defects [8, 9]. By shortening the injured limb through acute or chronic traction, the bone defects are temporarily eliminated, and then, the same length as the healthy side is obtained through gradual traction and lengthening. The docking site could be pressurized by the lengthening fixator at the shortening site. Most scholars agree that compared with traditional bone transport, ASR technique can shorten the EFI and reduce the complication incidence [6, 8, 9]. Yet, the application of ASR technique is limited due to the excessive traction of nerves, vessels and the soft tissue conditions. In addition, the occurrence of infection-related complications also puzzles some patients treated by ASR technique.

Calcium sulfate as an antibiotic carrier has been widely used in clinic and achieved satisfactory clinical effects. Calcium sulfate has good biocompatibility and degradability and does not need to be removed by a secondary surgery [10–12]. The antibiotic-loaded calcium sulfate is filled into the bone defect site after osteotomy, which can continuously release antibiotics and eliminate residual pathogenic bacteria. Meanwhile, it can effectively prevent local soft tissue insertion and degrade gradually. It is beneficial to patients using this technique. However, it has not been reported whether the ACSBT technique is superior to the ASR technique for the management of large segmental tibial defects. The purpose of this paper was to compare the clinical effects of ASR technique with ACSBT technique. We hypothesize that patients using ACSBT technique can obtain similar clinical effects as that of using ASR technique. ACSBT technique may be superior to ASR technique in controlling infection-related complications and improving the healing rate of docking site.

## Methods

The inclusion criteria are as follows: (1) patients with large segmental tibial defects after trauma; (2) age ranged from 18 to 65 years; (3) the bone loss was between 3 and 10 cm; and (4) patients with complete clinical and imaging data. The exclusion criteria are as follows: (1) large segmental tibial defects caused by tumor or congenital diseases; (2) patients younger than 18 or older than 65 years; (3) patients with bone loss less than 3 cm or more than 10 cm; (4) patients who cannot tolerate anesthesia or a surgery; and (5) lost patients.

General data: A total of 68 patients treated in our institution from June 2013 to June 2018 were collected retrospectively. There were 53 males and 15 females. The age ranged from 19 to 63 years. In ASR group, there were 32 patients, while ACSBT group concluded 36. There were 15 cases caused by acute trauma and 17 by chronic osteomyelitis in ASR group. ACSBT group contained 16 cases suffering from acute trauma and 20 by chronic osteomyelitis. All operations were performed by the same group of senior surgeons. This study has been approved by the ethics committee of our hospital. The informed consent of all selected patients has been achieved.

Preoperative treatment: Blood samples were drawn before operation to detect erythrocyte sedimentation rate (ESR), C-reactive protein (CRP), white blood cell (WBC) count, etc. All patients underwent thorough debridement in the first stage. During debridement, all sequestrum and completely free bone blocks shall be removed, and the two broken ends would be trimmed and leveled. If necessary, segmental resection could be performed. Patients in ASR group underwent acute shortening after debridement. Then, soft tissue defects were repaired by direct suture or skin grafting. In ACSBT group, skin grafting or a flap surgery was carried out to repair soft tissue defects as needed. After soft tissues healed and infection-related indexes returned to normal, different Ilizarov techniques were performed for bone reconstruction.

**Fig. 1:**
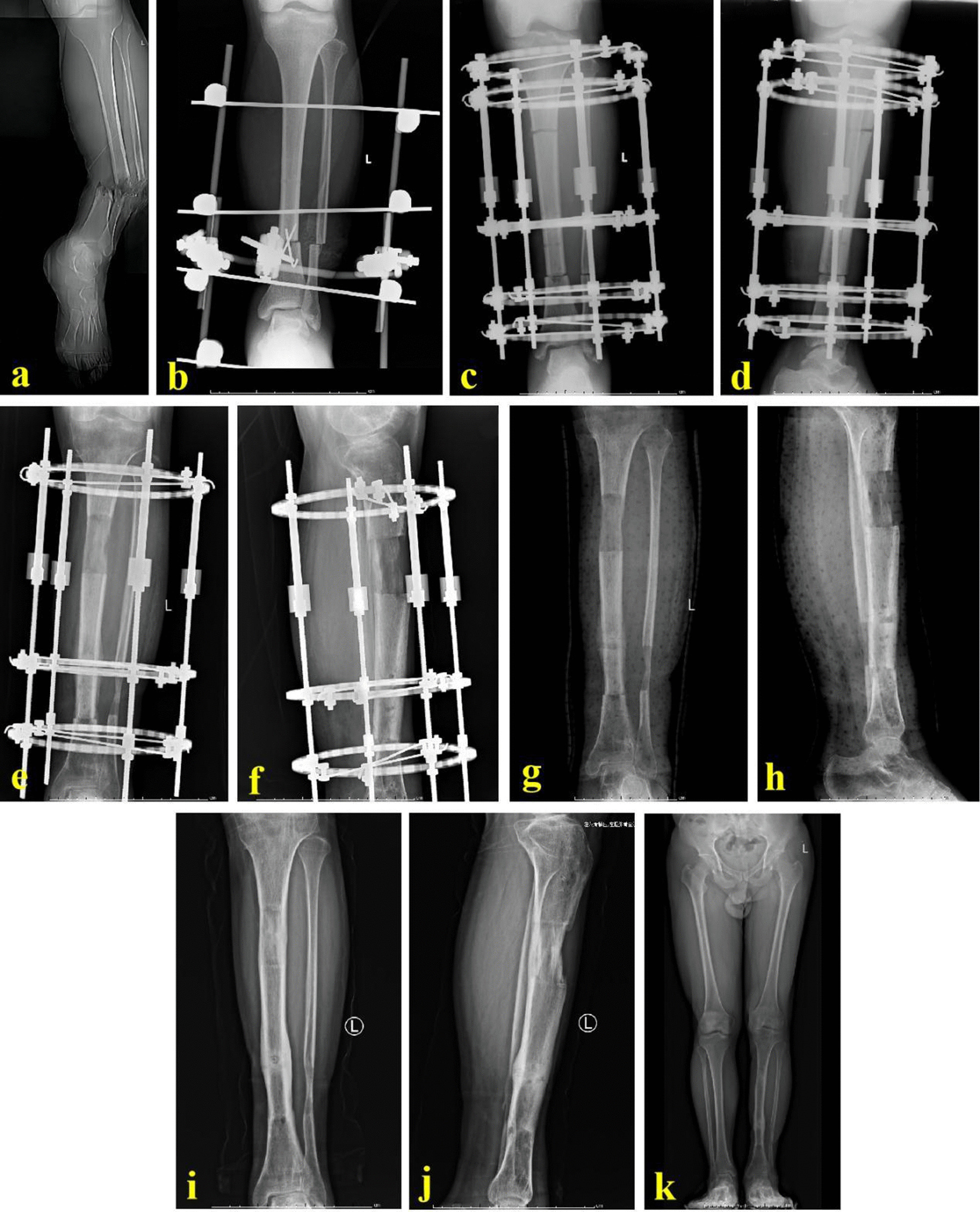
A 30-year-old male was successfully treated by ASR technique. **a** The patient suffered from a severe open fracture of the tibia; **b** after thorough debridement, acute shortening and temporary external fixation were performed. The tibial defects were 7.5 cm; **c**, **d** the Ilizarov annular lengthening frame was installed, and osteotomy was performed; **e**, **f** bone lengthening was successfully applied to restore the limb length; **g**, **h** X-ray images after removing the external frame; and **i**–**k** X-ray images at one year after removing the annular frame. ASR stands for acute shortening and re-lengthening

Operation steps: For ASR group, the two broken ends were shortened directly after thorough debridement. At this time, surgeons should closely observe circulation of the injured limb. In case of circulation disorders, the shortened length was extended by one centimeter and then circulation was observed continuously. When the circulation improved, the residual bone defects were shortened at a speed of 2–3 mm/d within the next 2 weeks until the bone defect ends were not exposed. If the fibula was intact, it should be cut off in the same wounds and the same length as the tibial defects was removed. Surgeons should ensure that the truncated part of the fibula was more than 6 cm above the ankle joint plane. Then, the wounds could heal naturally or by skin grafting. Cross Kirschner wires were inserted at the docking site, and the injured limb was temporarily fixed by an external fixator. After the wounds healed, and infection-related indexes returned to normal, the temporary fixator was removed. Meanwhile, the Kirschner wires were pulled out. The injured limb was maintained in the center of the annular lengthening frame. Parallel to the knee and ankle joint plane, the proximal and distal tibia were fixed, respectively. Low-energy osteotomy was performed at the proximal tibia. The limb axis and rotation were checked by an image intensifier. Bone lengthening began one week after operation, with an initial speed of one millimeter per day. Attention was paid to the circulation and limb feeling. If sensory or circulation disorders were observed, the lengthening was slowed down or even stopped until the disorders were eliminated. When the X-ray images showed that both lower limbs were equal in length, the lengthening stopped. The injured limb fully loaded to accelerate the consolidation. When the docking site firmly healed and the consolidation completed, the lengthening frame would be removed. A typical case is shown in Fig. 1.

**Fig. 2:**
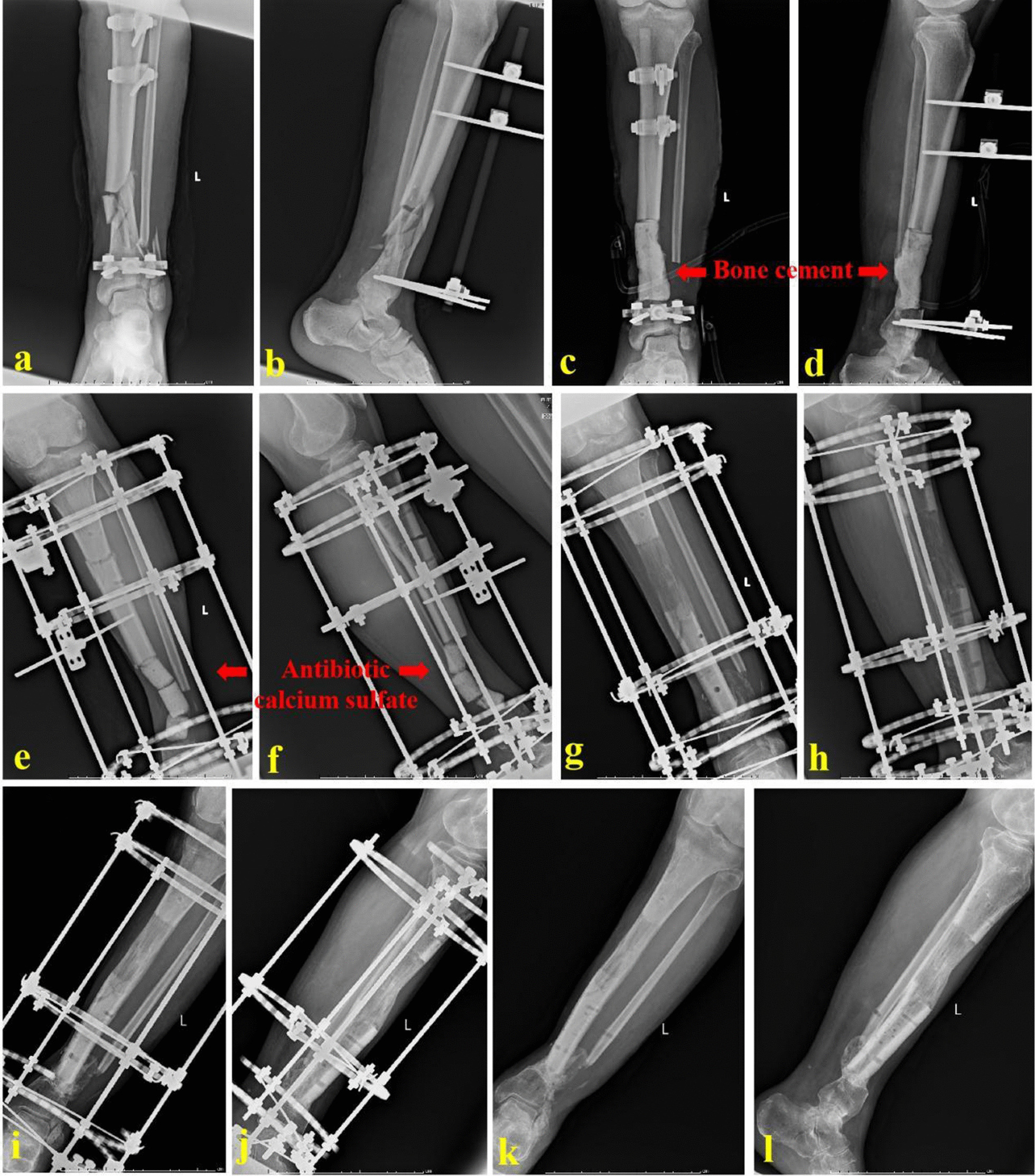
A 52-year-old male was successfully treated by ACSBT technique. **a**, **b** The patient suffered from a severe open fracture and infection of the tibia; **c**, **d** after thorough debridement, bone cement was filled into the bone defect site. The tibial defects were 9.0 cm; **e**, **f** the Ilizarov annular transport frame was installed, and antibiotic-loaded calcium sulfate was inserted; **g**–**j** ACSBT technique was successfully applied to repair the tibial defects; and **k**, **l** X-ray images at one year after removing the annular frame. ACSBT stands for antibiotic calcium sulfate-loaded bone transport

For ACSBT group, one-stage thorough debridement was performed, and specimens were taken for bacterial culture. The bone defects were first filled with regular bone cement and temporarily fixed by an external fixator. Soft tissue defects were repaired by skin grafting or a local flap. After the wounds healed and infection-related indexes returned to normal, the bone transport frame was installed. With the bone defect site as the center, an anterolateral longitudinal incision was taken. The bone defect site was exposed and regular bone cement was taken out. Debridement was performed again, and the fibrous scar tissues and sclerotic bones at both ends were removed. The initial limb length was maintained. Good alignment and rotation of the injured limb were ensured. Attention should be paid to keeping the foot in the neutral position. Then, the proximal, distal ends and transport segment were fixed, respectively. An external fixator for the foot and ankle was added if necessary. According to the bone defect size, antibiotic-loaded calcium sulfate was prepared (proportion: calcium sulfate 7.5 g + vancomycin 0.5 g + gentamicin injection 3 ml). In order to achieve the best anti-inflammatory effects, the loaded antibiotics should be adjusted according to the drug sensitivity test in time. Then, it was shaped into small cylinders and filled into the bone defect site. Low-energy osteotomy was performed at the metaphysis. The bone transport process was similar to that of bone lengthening. When the transport step completed, the docking site was pressured to promote healing. After the consolidation finished, the transport frame was removed. A typical case is shown in Fig. 2.

Postoperative treatment: Anti-inflammatory treatment was performed for 6 weeks according to the results of bacterial culture and drug sensitivity test. Intravenous antibiotics were used during hospitalization, and oral antibiotics could be used after discharge. X-ray films were rechecked every two weeks after operation. If axial deviation was found, it shall be handled in time. After operation, the pin-tracts were actively cared for every day to prevent infection. The tightness of all the screws was checked once a week, and the loosened screws were tightened in time. Functional exercises were carried out after operation. This could prevent joint stiffness and foot drop. Patients were followed through outpatient, telephone and WeChat platforms.

Observation indexes: The EFT and EFI were compared between the two groups, and the bone defect healing and limb functions were evaluated according to the ASAMI criteria [13, 14]. After evaluating the bone defect healing, infection, deformity and limb length difference, the bone results were calculated and divided into excellent, good, fair and poor. The limb function results were based on five criteria: (1) significant claudication; (2) stiffness of the knee or ankle (loss of full extension of the knee or extension or back extension of the ankle is more than 15° compared with the normal contralateral ankle); (3) soft tissue sympathetic dystrophy; (4) pain that interferes with activity or sleep; and (5) inactivity due to injury or inability to resume daily activities. The limb function results were also divided into excellent, good, fair and poor. Complications were classified according to Paley classification, including problems, obstacles and sequelae [14].

Statistical analysis: All statistical analyses were performed using SPSS23.0 software. Measurement data were expressed as mean ± standard deviation. Unpaired *t* test was used for comparisons between the two groups. Count data were analyzed using *χ*^2^ test. The *p* value of < 0.05 indicated a statistically significant difference.

## Results

### Comparison of demographic information

As shown in Table 1, the mean age in ASR and ACSBT group was 36 ± 9 years and 37 ± 8 years, respectively. There were 26 males and 6 females in ASR group, while 27 males and 9 females in ACSBT group. The mean bone loss in ASR and ACSBT group was 6.5 ± 1.8 cm and 7.1 ± 1.5 cm. The mean BMI was 25.6 ± 2.3 kg/m^2^ and 24.7 ± 2.8 kg/m^2^, respectively. There were 15 cases caused by acute trauma and 17 by chronic osteomyelitis in ASR group. ACSBT group contained 16 cases suffering from acute trauma and 20 by chronic osteomyelitis. The preoperative ESR was 15.5 ± 14.2 mm/h in ASR group and 17.1 ± 15.4 mm/h in ACSBT group, respectively; CRP was 12.78 ± 9.07 ng/L and 13.12 ± 9.25 ng/L, respectively; and WBC count was (8.19 ± 3.02) × 10^9^/L and (7.65 ± 3.84) × 10^9^/L, respectively. The average follow-up time of ASR and ACSBT group was 31 ± 4 months and 29 ± 5 months. There was no significant difference in demographic information between the two groups (*p* > 0.05).

**Table 1:** Demographic information of the two groups

Variable	ASR group (*n* = 32)	ACSBT group (*n* = 36)	*χ*^2^/*t*	*p* value
Age (year)	36 ± 9	37 ± 8	0.482	0.632
Gender (M/F)	26/6	27/9	0.385	0.535
*Etiology*			0.040	0.841
Acute trauma	15	16		
Osteomyelitis	17	20		
Bone loss (cm)	6.5 ± 1.8	7.1 ± 1.5	1.483	0.143
BMI (kg/m^2^)	25.6 ± 2.3	24.7 ± 2.8	1.454	0.151
ESR (mm/h)	15.5 ± 14.2	17.1 ± 15.4	0.446	0.657
CRP (ng/L)	12.78 ± 9.07	13.12 ± 9.25	0.153	0.879
WBC count (× 10^9^/L)	8.19 ± 3.02	7.65 ± 3.84	0.648	0.519
Follow-up (month)	31 ± 4	29 ± 5	1.830	0.072

ASR, acute shortening and re-lengthening; ACSBT, antibiotic calcium sulfate-loaded bone transport; BMI, body mass index; ESR, erythrocyte sedimentation rate; CRP, stands for C-reactive protein; and WBC, white blood cell

### Evaluation and comparison of clinical indexes

As shown in Table 2, the mean EFT of ASR and ACSBT group was 9.2 ± 1.8 months and 10.1 ± 2.0 months, respectively. The mean EFI in ASR and ACSBT group was 1.5 ± 0.2 month/cm and 1.4 ± 0.3 month/cm. According to the ASAMI criteria, in ASR group bone defect healing was excellent in 22 cases, good in 7 cases and fair in 3 cases. In ACSBT group, it was excellent in 23 cases, good in 11 cases and fair in 2 cases. In ASR group, the limb function was excellent in 15 cases, good in 7 cases and fair in 10 cases, while it was excellent in 14 cases, good in 9 cases and fair in 13 cases for ACSBT group. There was no significant difference in EFI, bone defect healing and limb functions between the two groups (*p* > 0.05).

**Table 2:** Clinical evaluation of the two groups

Variable	ASR group (*n* = 32)	ACSBT group (*n* = 36)	*χ*^2^/*t*	*p* value
EFT (month)	9.2 ± 1.8	10.1 ± 2.0	1.953	0.055
EFI (month/cm)	1.5 ± 0.2	1.4 ± 0.3	1.633	0.108
*Bone defect healing score*				
Excellent	22	23	0.179	0.672
Good	7	11	0.656	0.418
Fair	3	2	0.019	0.891
*Limb function score*				
Excellent	15	14	0.442	0.506
Good	7	9	0.092	0.762
Fair	10	13	0.179	0.672

ASR, acute shortening and re-lengthening; ACSBT, antibiotic calcium sulfate-loaded bone transport; EFT, external fixation time; and EFI, external fixation index

### Comparison of complications between the two groups

As shown in Table 3, complications were compared and categorized based on Paley classification. In ASR group, 18 problems were encountered, including 13 cases of Grade-II pin-tract infection, and 5 cases for transient loss of joint movement. Patients suffering from Grade-II pin-tract infection were given oral antibiotics and dressing. Those with transient loss of joint movement were guided to perform functional exercises. Twenty-seven obstacles were observed in ASR group, comprising 14 cases of Grade-III pin-tract infection, three cases of deep infection, four cases of docking site nonunion, one for axial deviation and five for joint stiffness. Grade-III pin-tract infection was managed by removing the infected pins and re-inserting a new one. Three cases with deep infection were treated by thorough debridement again and were given systemic antibiotics. Those with docking site nonunion were managed by bone grafting and internal fixation. The one with axial deviation was treated by adjusting the external frame. The number of sequelae in ASR group was 4. Patients suffering from sequelae rejected further surgeries.

**Table 3:** Comparison of complications between the two groups

Variable	ASR group (*n* = 32)	ACSBT group (*n* = 36)	*χ*^2^/*t*	*p* value
*Problems*				
Grade-II pin-tract infection	13	5	6.222	0.013
Aseptic exudation	0	7	4.990	0.025
Transient loss of joint movement	5	4	0.036	0.849
*Obstacles*				
Grade-III pin-tract infection	14	6	5.985	0.014
Deep infection	3	0	1.658	0.198
Docking site nonunion	4	5	0.036	0.849
Axial deviation	1	3	0.156	0.693
Joint stiffness	5	4	0.036	0.849
Soft tissue invagination	0	1	–	–
*Sequelae*				
Foot drop	4	3	0.027	0.869
Total	49	38		
Number of complications per patient	1.5 ± 0.6	1.1 ± 0.2	3.598	0.001

ASR, acute shortening and re-lengthening; ACSBT, antibiotic calcium sulfate-loaded bone transport

In ACSBT group, 16 problems were encountered, including 5 patients for Grade-II pin-tract infection, 7 for aseptic exudation and 4 for transient loss of joint movement. Patients suffering from aseptic exudation were managed by regular dressing. Nineteen obstacles were observed, comprising 6 patients for Grade-III pin-tract infection, 5 for docking site nonunion, 3 for axial deviation, 4 for joint stiffness and one for soft tissue invagination. The patient with soft tissue invagination was treated by surgical release. Three patients developed foot drop. Treatment of these complications was similar to that of ASR group. The mean number of complications per patient was 1.5 ± 0.6 and 1.1 ± 0.2 in ASR and ACSBT group, respectively (*p* < 0.05).

## Discussion

Segmental bone defects are one of the most challenging diseases faced by trauma surgeons, especially when accompanied by infection and soft tissue loss. These patients usually suffer a long and complex treatment cycle. Patients and their families face a great physical and mental burden. The main treatment methods for large segmental bone defects include autologous bone transplantation, Ilizarov technique, Masquelet technique, vascularized fibula transplantation and segmental prostheses [1, 5, 15–18]. Among them, Ilizarov technique is one of the most effective methods.

Several scholars have compared ASR technique with bone transport for the treatment of large segmental bone defects. Thakeb et al. [8] compared these two techniques in post-traumatic tibial nonunion patients with composite bone and soft-tissue defects. They found that the ASR technique may be superior because it had a significantly lower EFI. But ASR technique was limited by the defect size and the surrounding soft tissue conditions. Sen et al. [19] introduced a new modified technique of ASR using a monolateral external fixator combined with a retrograde intramedullary nail. They also compared its results with the classic Ilizarov bone transport for the management of infected nonunion of the distal femur with bone loss. Their results showed that the modified technique of ASR may confer greater patients’ satisfaction because of shorter EFI. In essence, this was a hybrid lengthening technique, or bone lengthening over an intramedullary nail. Although there were no patients suffering from severe infection recurrence in Sen’s study, the number of cases included in their study was small. Once infection recurrence occurs in the case of internal and external hybrid transport/lengthening, the results may be disastrous. This will not only increase the number of operations, but also greatly increase medical expenses. Sigmund et al. [9] performed a single-center prospective study with a standardized treatment protocol between ASR and bone transport technique. They found that all patients required an unplanned procedure in bone transport group, while less revision surgeries were needed for ASR group. Tetsworth et al. [6] included 42 patients of infected tibial nonunion with segmental bone loss in a retrospective comparative study. Based on their results, ASR technique demonstrated a lower complication incidence and a slightly better radiographic outcome. The above scholars have discussed both the advantages and disadvantages for bone transport and ASR technique. In terms of complications and EFI, the ASR technique displays slightly better effects than bone transport. However, the ASR technique still has some shortcomings. For example, in the face of bone loss longer than 10 cm, the ASR technique may be difficult to implement smoothly.

Patients with segmental bone defects are usually accompanied by large skin and soft tissue defects, especially those caused by chronic osteomyelitis. Due to multiple operations, extremely poor local skin conditions and circulation, deep infection is hard to eradicate by systemic antibiotics, and the antibacterial effects of systemic antibiotics are limited [20]. Therefore, scholars use different antibiotic carrier stents for local administration [21, 22]. Antibiotic-loaded calcium sulfate is an absorbable local antibiotic sustained-release system with good drug loading properties. Its advantages include more accurate positioning, higher local antibiotic concentrations, fewer side effects and longer treatment time [22]. Previous studies have shown that calcium sulfate loaded with antibiotics was effective for the treatment of bone infection [23, 24]. For patients with large bone defects caused by an open fracture or chronic osteomyelitis, the rational and effective application of antibiotics is essential. In our study, antibiotic-loaded calcium sulfate was filled into the bone defect site when bone transport technique was applied. Our results showed that the infection-related complication incidence in ACSBT group was lower than that in ASR group, including pin-tract infection and deep infection. In ASR group, only systemic antibiotics were used. However, patients in ACSBT group were given local and systemic antibiotics simultaneously. Therefore, it was more effective in controlling infection. This would also reduce the unplanned surgeries due to the management of infection-related complications.

Docking site nonunion is a common complication in bone transport [1, 5, 6, 8, 9]. It often requires additional surgical intervention, such as bone graft with internal fixation and tissue-engineered strategies [25, 26]. In our study, there were 4 cases suffering from docking site nonunion in ASR group and 5 cases in ACSBT group. The complication incidence was roughly the same. Calcium sulfate loaded with antibiotics could play a good space occupying role locally. This could avoid soft tissue insertion during bone transport process. With the progress of bone transport, the docking site is getting closer, and calcium sulfate is constantly squeezed and degraded. This is similar to the fact that both ends of bone defects are bridged together by antibiotic calcium sulfate carriers. This also reduces additional operations and time in external fixator due to the treatment of docking site nonunion.

In addition, our results showed that the incidence of axial deviation and aseptic exudation in ACSBT group was higher than that in ASR group. Due to limb weight-bearing, daily adjustment and no contact at the bone defect site, the axis would change gradually. If the patient did not recheck in time, it might not be able to find small deviation. With time accumulation, there would be obvious axial deviation. Finally, an additional surgery might be required to correct the axial deviation. This may be why there were more patients suffering from axial deviation in ACSBT group. Moreover, soft tissues around tibia are relatively weak, and it will become weaker after trauma. The aseptic exudation happens easily around the weak and injured soft tissues. It was reported that the aseptic exudation incidence after calcium sulfate application was 4–51% [19, 20]. This serous exudation gradually alleviates with the absorption of calcium sulfate. Generally, only local dressing is required for the management of aseptic exudation.

Our research still had some deficiencies. This study was a retrospective study in essence. There were differences caused by personal preferences and soft tissue conditions. Therefore, the conclusions in this study still need to be verified by further prospective randomized controlled studies. In addition, the number of patients in this study was limited and the follow-up time was relatively short. We will conduct a large sample size and long-term follow-up study in further research.

## Conclusions

Both techniques can be successfully used for the management of large segmental tibial defects after trauma. There was no significant difference in EFI, limb functions and bone defect healing between the two groups. Compared with ASR group, the complication incidence in ACSBT group was lower, especially the infection-related complications. Therefore, for patients with large segmental bone defects caused by infection or osteomyelitis, ACSBT technique could be the first choice.

## Data Availability

All data analyzed in this study have been provided in the manuscript.

## Abbreviations

ASR: Acute shortening and re-lengthening
ACSBT: Antibiotic calcium sulfate-loaded bone transport
EFT: External fixation time
EFI: External fixation index
ASAMI: The Association for the Study and Application of the Method of Ilizarov
BMI: Body mass index
ESR: Erythrocyte sedimentation rate
CRP: C-reactive protein
WBC: White blood cell
